# Carbon and nitrogen isotopic ratios of urine and faeces as novel nutritional biomarkers of meat and fish intake

**DOI:** 10.1007/s00394-012-0328-2

**Published:** 2012-03-10

**Authors:** Gunter G. C. Kuhnle, Annemiek M. C. P. Joosen, Catherine J. Kneale, Tamsin C. O’Connell

**Affiliations:** 1 Department of Food and Nutritional Sciences, University of Reading, Whiteknights, PO Box 226, Reading, RG6 6AP UK; 2 MRC Dunn Human Nutrition Unit, Wellcome Trust/MRC Building, Cambridge, UK; 3 MRC Centre for Nutritional Epidemiology in Cancer Prevention and Survival, Department of Public Health and Primary Care, University of Cambridge, Cambridge, UK; 4 Department of Archaeology and Anthropology, University of Cambridge, Cambridge, UK; 5 McDonald Institute for Archaeological Research, University of Cambridge, Cambridge, UK

**Keywords:** Stable isotope ratio, Dietary assessment, Nutritional biomarker, Fish, Meat

## Abstract

**Purpose:**

Meat and fish consumption are associated with changes in the risk of chronic diseases. Intake is mainly assessed using self-reporting, as no true quantitative nutritional biomarker is available. The measurement of plasma fatty acids, often used as an alternative, is expensive and time-consuming. As meat and fish differ in their stable isotope ratios, δ^13^C and δ^15^N have been proposed as biomarkers. However, they have never been investigated in controlled human dietary intervention studies.

**Objective:**

In a short-term feeding study, we investigated the suitability of δ^13^C and δ^15^N in blood, urine and faeces as biomarkers of meat and fish intake.

**Methods:**

The dietary intervention study (*n* = 14) followed a randomised cross-over design with three eight-day dietary periods (meat, fish and half-meat–half-fish). In addition, 4 participants completed a vegetarian control period. At the end of each period, 24-h urine, fasting venous blood and faeces were collected and their δ^13^C and δ^15^N analysed.

**Results:**

There was a significant difference between diets in isotope ratios in faeces and urine samples, but not in blood samples (Kruskal–Wallis test, *p* < 0.0001). In pairwise comparisons, δ^13^C and δ^15^N were significantly higher in urine and faecal samples following a fish diet when compared with all other diets, and significantly lower following a vegetarian diet. There was no significant difference in isotope ratio between meat and half-meat–half-fish diets for blood, urine or faecal samples.

**Conclusions:**

The results of this study show that urinary and faecal δ^13^C and δ^15^N are suitable candidate biomarkers for short-term meat and fish intake.

## Introduction

Red meat and fish consumption are both associated with changes in the risk of chronic diseases; whereas red meat is associated with an increased risk of cancer [30], fish consumption is often associated with a reduced risk of cardiovascular diseases [15] and diabetes [21], in particular because of its content of *n*-3 polyunsaturated fatty acids, but possibly also because of other compounds such as selenium [35]. The investigation of associations between disease risk and meat and fish intake in observational studies relies on accurate dietary information; however, most dietary assessment instruments rely to some extent on self-reporting that is prone to systematic bias [10, 11]. Statistical models developed to accommodate exposure measurement errors require at least two exposure assessments with unrelated measurement error, and this cannot be achieved using methods relying on self-reporting alone. However, nutritional biomarkers can provide such an assessment method [24]. Although several compounds have been proposed as candidate biomarkers for meat, for example, methyl histidine or carnitine, a true quantitative biomarker does not currently exist [6]. Metabolomics techniques have also been used to assess meat consumption, but these techniques require complex data analyses. Fish consumption is often assessed using either plasma or red blood cell (RBC) fatty acids, or environmental contaminants such as methyl mercury (MeHg) [35], whereas fatty acid analyses require complex analytical procedures and are often not suitable for large-scale epidemiological studies, environmental contaminants depend on the fishing area and fish species, thus introducing an additional bias.

An alternative biomarker for meat and fish intake is the ratio of naturally occurring stable isotopes of carbon (^13^C/^12^C ratio expressed as δ^13^C) and nitrogen (^15^N/^14^N ratio expressed as δ^15^N). These ratios are used extensively for dietary assessment in archaeological and ecological studies [26]; however, their application in nutritional epidemiology is very limited. The stable isotope composition of hair has been used as a biomarker of animal protein intake [22], and recently, O’Brien et al*.* have developed δ^15^N in red blood cells as a surrogate marker of eicosapentanoic acid (EPA) and docosahexaenoic acid (DHA) intake in a population of Yup’ik Eskimos [20]. This study showed a strong significant correlation between RBC δ^15^N and RBC EPA and DHA, and a weaker correlation with self-reported intake. However, this study was limited to a population with high habitual fish intake and relied on self-reporting for dietary assessment.

In this study, we investigate δ^13^C and δ^15^N of urine and faeces as candidate biomarkers for meat and fish intake; furthermore, we contrast this to the effect of short-term dietary changes on the stable isotope ratio in whole blood, which is a slow-turnover tissue. In a carefully controlled dietary intervention study, we have investigated associations between dietary meat and fish, and carbon and nitrogen isotope ratios in blood, faeces and 24-h urine samples.

## Subjects and methods

### Diet intervention studies

Dietary intervention studies were conducted as described previously [13, 14]. Subjects were recruited through local advertisement and completed a medical questionnaire before entering the study and only those in good health were accepted. Subjects received verbal and written information and signed a written consent form. All studies were approved by the Cambridge Local Research Ethics Committee. Fourteen volunteers (8 female, 6 male, age: 27 ± 7 years, BMI: 24 ± 5 kg/m^2^), free from diabetes and bowel disease, not taking any medication affecting the gastro-intestinal tract and not taking any nutritional supplements, were included in the study.

Participants lived in the volunteer suite of the MRC Dunn Human Nutrition Unit (Cambridge, UK), where all food was provided and all specimens collected and processed. Participants followed their normal daily routine but were only allowed to consume foods and drinks prepared by the diet technicians. Body weights were monitored throughout the study to ensure constant weight. Daily faecal samples were collected to determine mean transit time [4] and check compliance. Blood, faeces and 24-h urine for isotope ratio analysis were collected at the last day of each dietary intervention. Fasting venous blood was drawn into citrated blood tubes. Whole blood aliquots were snap frozen on dry ice and stored at −80 °C until analysis. Faecal samples were collected on dry ice, X-rayed to measure transit time markers and stored at −20 °C until analysis. Faecal homogenates were prepared by diluting approximately 40 g stool with ultrapure water (ratio 1:5) and homogenising the sample for 20 min in a stomacher (Colworth 3500, Seward Medical, Worthing, UK). The faecal homogenates were snap frozen on dry ice and stored at −20 °C until analysis.

Dietary intervention studies followed a randomised cross-over design; diets were provided as similar menus following a 3-day rotating schedule; each intervention lasted for at last eight days (Table 1). Duplicates of each diet were prepared, homogenised and stored at −20 °C until analysis. Energy intake of each participant was matched to estimated energy requirement [5] with 1-MJ standardised increments (shortbread or a combination of white bread, butter and marmalade) added to 8 MJ/d (female) or 10 MJ/d (males) basal diets. Except for meat and fish, all food ingredients were kept the same in the meat and fish intervention diets; in the vegetarian control diet, fat content was kept similar by exchanging protein for carbohydrates. Energy and macronutrient composition of the diets were calculated using DINER (Data Into Nutrients for Epidemiological Research) [34]. All participants completed the red meat and fish intervention; however, only 13 completed the half-meat–half-fish dietary intervention. In addition, four volunteers completed a vegetarian diet. Urine samples were available for all dietary periods and all participants, except for one participant on the half-meat–half-fish diet. Blood samples were only available for 12 participants on the red meat diet, 13 participants on the fish diet and 11 participants on the half-meat–half-fish, but none for participants on the vegetarian diet.

**Table 1:** Intervention diets

	Vegetarian	Meat	½ Meat/½ Fish	Fish
*n*	4	14	14	14
Energy^a^	9	9	9	9
Protein^b^	77 (14%)	123 (23%)	117 (22%)	111 (20%)
Carbohydrates^b^	321 (59%)	256 (48%)	254 (47%)	253 (47%)
Fat^b^	79 (31%)	79 (32%)	85 (34%)	91 (36%)
SFA^b^	33 (12%)	38 (16%)	37 (15%)	35 (14%)
MUFA^b^	23 (9%)	25 (11%)	28 (12%)	31 (12%)
PUFA^b^	14 (6%)	6 (2%)	10 (4%)	14 (6%)
*n*-*3* PUFA^b^	n/d^c^	1 (0.4%)	4 (2%)	8 (3%)
*n*-*6* PUFA^b^	n/d^c^	5 (2%)	5 (2%)	5 (2%)
Intervention diet (male volunteers; raw weight)				
Day 1 and 3 Lunch	–	Roast beef (113 g)^e^	Mackerel^d^ (50 g); Roast beef (56 g)	Mackerel^d^ (100 g)
Day 1 and 3 Dinner	–	Mince beef (213 g)	Salmon (138 g); Mince beef (106 g)	Salmon (275 g)
Day 2 Lunch	–	Mince beef (113 g)	Herring^d^ (50 g); Roast beef (56 g)	Herring^d^ (100 g)
Day 2 Dinner	–	Roast beef (213 g)^e^	Trout (138 g); Mince beef (106 g)	Trout (275 g)
Isotope ratio^f^ (mean ± S.E.), ‰				
δ^13^C	−25.7 ± 0.1	−26.1 ± 0.2	−25.4^‡^	−24.7 ± 0.3
δ^15^N	3.8 ± 1.1	6.5 ± 0.1	7.4^‡^	8.3 ± 0.3

*SFA* saturated fatty acids, *MUFA* monounsaturated fatty acids, *PUFA* polyunsaturated fatty acids

^a^In MJ/d, ^b ^in g/d (contribution to total energy in parentheses), ^c ^not determined, ^d ^from tin, rinsed, no brine, ^e ^cooked weight, ^f ^analysed from duplicate diets, ^‡ ^Calculated as 1:1 mixture of meat and fish diet

### Sample analysis

Duplicate diet samples were analysed as liquid homogenates representative of 24-h food intake for each of the three diets. Samples were lyophilised and weighed into tin capsules (0.8 mg per aliquot). Faecal samples were analysed as liquid homogenates; an aliquot of the liquid homogenate was pipetted into a tin capsule and lyophilised prior to analysis. Samples were analysed separately for carbon and nitrogen isotopic values, typically using 10–50 μl of faecal homogenate for carbon, and 30–220 μl of faecal homogenate for nitrogen isotopic analysis.

Urine was directly pipetted into a tin capsule containing a porous polymer adsorbent (Chromasorb^®^) and analysed separately for carbon and nitrogen isotopic values, typically using 10–50 μl of urine for carbon, and 40–140 μl of urine for nitrogen isotopic analysis. Whole blood samples (0.5 mL) were lyophilised and then weighed into tin capsules (ca. 0.8 mg). Diet, faecal and urine samples were all isotopically analysed in duplicate, whilst blood samples were run in triplicate.

Isotopic analyses were performed using a Costech (Valencia, CA) automated elemental analyser coupled in continuous-flow mode to a Thermo Finnigan MAT253 (Bremen, Germany) mass spectrometer at the Godwin Laboratory, Department of Earth Sciences, University of Cambridge. Stable isotope concentrations are measured as the ratio of the heavier isotope to the lighter isotope relative to an internationally defined scale, VPDB for carbon and AIR for nitrogen [12]. Isotopic results are reported as δ values (δ^13^C and δ^15^N) in parts per 1000 or ‘permil’ (‰) values, where δ*X* = [(*R*_sample_/*R*_Standard_) − 1] × 1000, where *R* is the ratio of heavy to light isotope (for both nitrogen and carbon). Based on replicate analyses of international and laboratory standards, measurement errors are less than ±0.2‰ for δ^13^C and δ^15^N.

### Statistical analysis

The sample size and distribution of data made it necessary to use nonparametric statistic tests. Differences in δ^13^C and δ^15^N between different diets were analysed using the Wilcoxon rank-sum test and Kruskal–Wallis test; the significance level was 0.05. As only four participants completed the vegetarian diet, these data were not included in the Kruskal–Wallis test. Power calculations were performed with G*Power 3.1.2 [8]. The sample size was sufficient to detect differences in δ^13^C and δ^15^N of 2.5% with a power (1 − β) of 0.8 at a significance level of α = 0.05, assuming a standard deviation of 2%. Data analysis was conducted in Stata11 for Mac (Sata Corp, College Station, Texas). Bivariate boxplots (bagplots, [28]) were prepared in R 2.12.1 [25].

## Results

**Fig. 1:**
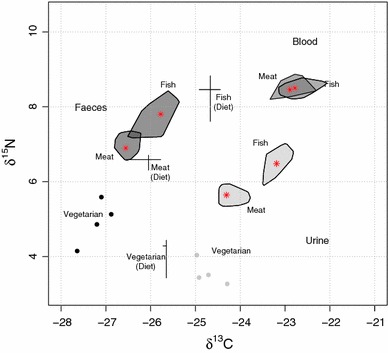
Isotope ratios (δ^13^C and δ^15^N) of intervention diets in faeces (*dark grey*), blood (medium) and urine (*light grey*). Dietary data is shown as median and inter-quartile range; data for blood, faecal samples and urine (except for vegetarian diet where individual results are shown) are shown as bagplot (50% of samples are within the *grey area*)

A summary of the results is shown in Fig. 1, and more detailed data are shown in Table 2. No statistically significant effect of the sequence of diets was observed. Whereas statistically significant differences were detected for both faecal and urinary δ^13^C and δ^15^N between diets (*p* < 0.001; Kruskal–Wallis test), no significant differences were found for blood samples. A pairwise comparison (Table 3) showed significant differences for δ^13^C and δ^15^N in urine and faeces samples between the vegetarian diet and all other diets, as well as between the fish diet and all other diets; the difference in isotope ratios in blood, faeces and urine between the half-fish–half-meat diet and the red meat diet was not statistically significant. There was no significant difference of δ^13^C and δ^15^N between men and women (Wilcoxon rank-sum test). Urinary δ^15^N was inversely correlated with age (Spearman’s ρ: −0.39, *p* < 0.01), weight (Spearman’s ρ: −0.42; *p* < 0.005) and BMI (Spearman’s ρ: −0.50; *p* < 0.001).

**Table 2:** Isotope ratios (δ^13^C and δ^15^N) of blood, faeces and urine (median and inter-quartile range)

Diet group	*n*	δ^13^C (‰)	δ^15^N (‰)
Blood^a^			
Fish	13	−22.7 (−23.0 to −22.6)	8.5 (8.4–8.6)
Meat/Fish	11	−23.0 (−23.2 to −22.6)	8.5 (8.3–8.7)
Meat	14	−22.8 (−23.1 to −22.5)	8.5 (8.3–8.7)
Vegetarian	–	–	–
Faeces^b^			
Fish	14	−23.2 (−23.4 to −22.9)	6.7 (6.3–6.8)
Meat/fish	13	−24.0 (−24.3 to −23.5)	5.7 (5.2–6.0)
Meat	14	−24.3 (−24.4 to −23.8)	5.6 (5.5–5.9)
Vegetarian	4	−24.8 (−25.0 to −24.5)	3.5 (3.4–3.8)
Urine^b^			
Fish	14	−25.8 (−26.2 to −25.5)	7.8 (7.3–8.1)
Meat/fish	13	−26.4 (−26.6 to −26.2)	7.0 (6.8–7.8)
Meat	14	−26.5 (−26.7 to −26.3)	6.9 (6.7–7.2)
Vegetarian	4	−27.2 (−27.4 to −27.0)	5.0 (4.5–5.4)

^a^No significant differences between diets (*p* > 0.6 for δ^13^C and δ^15^N, Kruskal–Wallis test)

^b^Significant differences between diets (*p* < 0.0005 for δ^13^C and δ^15^N, Kruskal–Wallis test)

**Table 3:** Pairwise comparison (Wilcoxon rank-sum test) of δ^13^C and δ^15^N following different diets

Diet	Specimen	Fish/meat		Red meat		Vegetarian	
		δ^13^C	δ^15^N	δ^13^C	δ^15^N	δ^13^C	δ^15^N
Fish	Blood	n. s.	n. s.	n. s.	n. s.	n. d.	
	Faeces	*p* < 0.005	*p* < 0.01	*p* < 0.0005	*p* < 0.0005	*p* < 0.005	*p* < 0.005
	Urine	*p* < 0.005	*p* < 0.05	*p* < 0.0005	*p* < 0.005	*p* < 0.005	*p* < 0.005
Fish/Meat	Blood			n. s.	n. s.	n. d.	
	Faeces			n. s.	n. s.	*p* < 0.05	*p* < 0.005
	Urine			n. s.	n. s.	*p* < 0.05	*p* < 0.005
Red Meat	Blood					n. d.	
	Faeces					*p* < 0.05	*p* < 0.005
	Urine					*p* < 0.05	*p* < 0.005

n. s.: not significant at significance level *p* = 0.05; n. d.: not determined

The offset between dietary isotope ratio and isotope ratio determined in specimens is shown in Table 4 for each diet. Whereas urine samples had a higher δ^13^C and lower δ^15^N than the respective diet, faecal samples showed a lower δ^13^C and δ^15^N for the meat and vegetarian diet, but only a lower δ^15^N for the fish diet. There was no significant difference between male and female participants, but in faecal samples, the δ^13^C offset from diet was positively correlated with BMI (Spearman’s ρ: 0.48; *p* < 0.005).

**Table 4:** Offset between δ^13^C and δ^15^N in diet and specimen (median and inter-quartile range)

	Blood		Faeces		Urine	
	δ^13^C (‰)	δ^15^N (‰)	δ^13^C (‰)	δ^15^N (‰)	δ^13^C (‰)	δ^15^N (‰)
Fish	−2.0 (−2.1 to −1.7)	−0.3 (−0.4 to −0.1)	1.1 (0.8–1.5)	0.4 (0.2–1.0)	−1.5 (−1.8 to −1.3)	1.6 (1.5–2.0)
Meat	−3.3 (−3.6 to −3.0)	−1.9 (−2.1 to −1. 8)	0.4 (0.2–0.6)	−0.4 (−0.6 to −0.1)	−1.8 (−2.2 to −1.7)	1.0 (0.7–1.1)
Vegetarian			1.5 (1.3–1.7)	−1.2 (−2.1 to −1.8)	−0.9 (−1.2 to −0.8)	0.3 (0.0–0.5)

Offset calculated as δ^13^C_diet_ − δ^13^C_specimen_ and δ^15^N_diet_ − δ^15^N_specimen_

## Discussion

In this study, we have investigated the feasibility of δ^13^C and δ^15^N in different specimens as biomarkers for short-term meat and fish intake. The results from this study provide information about the validity of δ^13^C and δ^15^N as nutritional biomarkers of meat and fish intake. The foodstuffs used are commonly consumed in North–West Europe and therefore represent typical dietary sources of meat and fish, although the amounts of meat and fish in the intervention diet were higher than the average consumption in the UK, in particular for fish (average daily consumption by men in the UK: 217 g (meat); 8 g (oily fish); National Diet and Nutrition Survey 2008/9 [19]). Studies with diets comparable to the habitual intake of meat and fish are required for further validation of these markers.

In this study, we found significant differences in δ^13^C and δ^15^N between dietary periods in faeces and urine; however, no significant differences in blood. This is unsurprising given the half-life and slow turnover of the main source of protein, red blood cells, whose half-life is considerably longer than the time of the dietary intervention. This is also an explanation for the observed offsets between isotope ratios in diet and blood. The ranges of δ^13^C and δ^15^N determined in blood are comparable to results from 406 patients on a normal habitual diet at the Johns Hopkins Medical Institutions (δ^13^C: clot; −16.4 to −23.4‰; serum: −15.8 to −23.2‰; δ^15^N: clot: 7.7–10.5‰; serum: 6.3–8.7‰) [16]; the δ^15^N observed was also within the range found in red blood cells of Yup’ik Eskimos [36].

Faecal samples are not routinely used as specimens for nutritional biomarkers in current studies, whereas coprolites are often used to assess prehistoric diets [27], and faecal samples are now used as isotopic markers of diet in ecological studies [3]. The results from this study show significant differences in carbon and nitrogen isotope ratios between different diets, suggesting that faecal samples can be used to determine differences in the amount of meat and fish consumed. In all diets, faecal samples contained less ^13^C than the diet; however, only following a fish diet faeces were also depleted in ^15^N, whereas the amount of ^15^N was higher following meat and in particular vegetarian diets. This pattern of nitrogen isotopic enrichment of faeces relative to diet has been observed in controlled feeding studies of animals [31]. The depletion in ^15^N of faeces for individuals on the fish diet might be due to the short duration of the dietary period and the difference in fish and subsequently ^15^N intake compared with participants’ habitual diets. The observed correlation between BMI and faecal δ^13^C could be explained by the higher amount of carbohydrates consumed by participants with a higher energy requirement, although the data available do not allow to investigate this further.

Quantified urinary nitrogen is commonly used as biomarker of protein intake [1] as most nitrogen from protein metabolism is excreted in urine. As nutritional biomarker, it reflects short-term intake of protein, and therefore it can be expected that the ratio of nitrogen isotopes will also reflect short-term dietary intake; this is confirmed by results from this study. Previous studies have shown that urine is depleted in ^15^N compared to body tissues [9, 31], and differences between urinary and blood δ^15^N in this study confirm these results. Urinary nitrogen is also depleted in ^15^N compared to diet, and this depletion is more pronounced for diets with a high δ^15^N such as fish. Conversely, δ^13^C is significantly increased compared to diet in all diet groups.

Meat, fish and plant based foods are not isotopically homogenous categories. The isotopic values of meat and fish are dependent on the animal’s diet and the location and environment in which they live, and the isotopic values of plants vary depending on the climate and environment in which they are grown. Isotopic variation within and between ecosystems has been widely documented in archaeological and ecological studies over many years [2, 7, 32, 33]. Thus one expects and observes inherent isotopic variations within food sources, depending on their origin. However, we have shown that in a population fed on a diet of plants, meat and fish typical for North-West Europe, significant isotopic differences in dietary intake translate to significant differences in faecal and urinary isotopic values even after a short-term dietary intervention. Although the diet used in this study contained only beef and not other types of meat, previous studies have shown that in particular beef and pork show similar stable isotope ratios [18, 29] and it is therefore likely that these results are applicable to diets containing different types of meat.

In summary, we show that urinary and faecal δ^13^C and δ^15^N are suitable candidate biomarkers for short-term meat and fish intake. The statistically significant isotopic differences in urine and faeces between those on meat and fish diets are sufficient to distinguish between consumers of different diets. The results also suggest that it is possible to identify vegetarian diets though the sample size was insufficient to provide more accurate data. Although it was possible to distinguish between diets containing only fish and those containing both meat and fish, it was not possible to distinguish between diets containing meat and diets containing both meat and fish. This might be a limitation of this biomarker, or of the length of time of the dietary intervention, but further studies are required to refine these results. In contrast to faeces and urine, blood δ^13^C and δ^15^N did not change sufficiently between diets on the timescale of this study to detect significant differences. This can be explained by the half-life of red blood cells and other major protein sources in blood, which exceeded the time of the intervention diet, as has been observed in previous studies [17, 23]. Whereas urinary and faecal δ^13^C and δ^15^N are therefore suitable biomarkers for short-term meat and fish intake, δ^13^C and δ^15^N in blood are more suitable as biomarkers of habitual or long term intake of meat and fish.

Urine and faecal samples are ideal specimens for dietary assessment with biomarkers, since they can be collected non-invasively, and their stable isotope ratios can be analysed easily without laborious sample preparation and data processing, in contrast to fatty acid analysis, for example. Furthermore, they can be determined in stored or archived samples, enabling comparisons with other existing data sets, such as total N intake. Ease of sampling, storage and analysis makes stable isotope ratio analysis in these specimens particularly useful for large-scale epidemiological studies.

## References

[1] Bingham SA (2003). Urine nitrogen as a biomarker for the validation of dietary protein intake. J Nutr.

[2] Bowen GJ (2010). Isoscapes: spatial pattern in isotopic biogeochemistry. Annu Rev Earth Planet Sci.

[3] Cordon D, Cordon J, Lee-Thorp JA, Sponheimer M, De Ruiter D, Sealy J, Grant R, Fourie N (2007). Diets of savanna ungulates from stable carbon isotope composition of faeces. J Zool.

[4] Cummings JH, Wiggins HS (1976). Transit through the gut measured by analysis of a single stool. Gut.

[5] Department of Health (UK) (1991) Dietary reference values for food, energy and nutrients for the United Kingdom (Report on health and social subjects No. 41). In: London 1961974

[6] Dragsted LO (2010). Biomarkers of meat intake and the application of nutrigenomics. Meat Sci.

[7] Dufour E, Bocherens H, Mariotti A (1999). Palaeodietary implications of isotopic variability in Eurasian Lacustrine fish. J Archaeol Sci.

[8] Erdfelder E, Faul F, Buchner A (1996). GPOWER: a general power analysis program. Behav Res Methods Instrum Comput.

[9] Fuller BT, Fuller JL, Sage NE, Harris DA, O’Connell TC, Hedges REM (2004) Nitrogen balance and delta15N: why you’re not what you eat during pregnancy. Rapid Commun Mass Spectrom 18:2889–2896. doi:10.1002/rcm.1708 10.1002/rcm.1708 15517531

[10] Hebert JR, Clemow L, Pbert L, Ockene IS, Ockene JK (1995). Social desirability bias in dietary self-report may compromise the validity of dietary intake measures. Int J Epidemiol.

[11] Hebert JR, Ma Y, Clemow L, Ockene IS, Saperia G, Stanek EJ, Merriam PA, Ockene JK (1997). Gender differences in social desirability and social approval bias in dietary self-report. Am J Epidemiol.

[12] Hoefs J (1997). Stable Isotope Geochemistry.

[13] Joosen AMCP, Kuhnle GGC, Aspinall SM, Barrow TM, Lecommandeur E, Azqueta A, Collins AR, Bingham SA (2010). Effect of processed and red meat on endogenous nitrosation and DNA damage. Carcinogenesis.

[14] Joosen AMCP, Lecommandeur E, Kuhnle GGC, Aspinall SM, Kap L, Rodwell SA (2010). Effect of dietary meat and fish on endogenous nitrosation, inflammation and genotoxicity of faecal water. Mutagenesis.

[15] Konig A, Bouzan C, Cohen JT, Connor WE, Kris-Etherton PM, Gray GM, Lawrence RS, Savitz DA, Teutsch SM (2005). A quantitative analysis of fish consumption and coronary heart disease mortality. Am J Prev Med.

[16] Kraft R, Jahren A, Saudek C (2008). Clinical-scale investigation of stable isotopes in human blood: delta13C and delta15N from 406 patients at the Johns Hopkins Medical Institutions. Rapid Commun Mass Spectrom.

[17] Martinez Del Rio C, Anderson-Sprecher R (2008). Beyond the reaction progress variable: the meaning and significance of isotopic incorporation data. Oecologia.

[18] Minagawa M (1992). Reconstruction of human diet from d13C and d15N in contemporary Japanese hair: a stochastic method for estimating multi-source contribution by double isotopic tracers. Appl Geochem.

[19] National Diet and Nutrition Survey—Headline results from Year 1 of the Rolling Programme. In: Bates B, Lennox A, Swan G (eds) (2009)

[20] O’Brien DM, Kristal AR, Jeannet MA, Wilkinson MJ, Bersamin A, Luick B (2009). Red blood cell delta15N: a novel biomarker of dietary eicosapentaenoic acid and docosahexaenoic acid intake. Am J Clin Nutr.

[21] Patel PS, Sharp SJ, Luben RN, Khaw K-T, Bingham SA, Wareham NJ, Forouhi NG (2009). Association between type of dietary fish and seafood intake and the risk of incident type 2 diabetes. Diabetes Care.

[22] Petzke K, Boeing H, Klaus S, Metges C (2005). Carbon and nitrogen stable isotopic composition of hair protein and amino acids can be used as biomarkers for animal-derived dietary protein intake in humans. J Nutr.

[23] Phillips DL, Eldridge PM (2006). Estimating the timing of diet shifts using stable isotopes. Oecologia.

[24] Prentice RL, Sugar E, Wang CY, Neuhouser M, Patterson R (2002). Research strategies and the use of nutrient biomarkers in studies of diet and chronic disease. Public Health Nutr.

[25] R Development Core Team (2009) R: A language and environment for statistical computing. Vienna, Austria

[26] Richards MP, Pettitt PB, Trinkaus E, Smith FH, Paunović M, Karavanić I (2000). Neanderthal diet at Vindija and Neanderthal predation: the evidence from stable isotopes. Proc Natl Acad Sci USA.

[27] Riley T (2008). Diet and seasonality in the Lower Pecos: evaluating coprolite data sets with cluster analysis. J Archaeol Sci.

[28] Rousseeuw PJ, Ruts I, Tukey JW (1999). The bagplot: a bivariate boxplot. Am Stat.

[29] Schoeller DA, Minagawa M, Slater R, Kaplan IR (1986) Stable isotopes of carbon, nitrogen and hydrogen in the contemporary north American human food web. Ecology of Food and Nutrition 1986:3

[30] Sinha R, Cross AJ, Graubard BI, Leitzmann MF, Schatzkin A (2009). Meat intake and mortality: a prospective study of over half a million people. Arch Internal Med.

[31] Sponheimer M, Robinson T, Roeder B, Ayliffe L, Passey B, Cerling T, Dearing D, Ehleringer J (2003). An experimental study of nitrogen flux in llamas: is ^14^N preferentially excreted?. J Archaeol Sci.

[32] Stevens RE, Hedges REM (2004). Carbon and nitrogen stable isotope analysis of northwest European horse bone and tooth collagen, 40,000 BP-present: palaeoclimatic interpretations. Quat Sci Rev.

[33] Valenzuela LO, Chesson LA, O’Grady SP, Cerling TE, Ehleringer JR (2011). Spatial distributions of carbon, nitrogen and sulfur isotope ratios in human hair across the central United States. Rapid Commun Mass Spectrom.

[34] Welch AA, McTaggart A, Mulligan AA, Luben RN, Walker N, Khaw K-T, Day NE, Bingham SA (2001). DINER (Data Into Nutrients for Epidemiological Research)—a new data-entry program for nutritional analysis in the EPIC-Norfolk cohort and the 7-day diary method. Public Health Nutr.

[35] Wennberg M, Bergdahl IA, Hallmans G, Norberg M, Lundh T, Skerfving S, Strömberg U, Vessby B, Jansson J-H (2011). Fish consumption and myocardial infarction: a second prospective biomarker study from northern Sweden. Am J Clin Nutr.

[36] Wilkinson MJ, Yai Y, O’Brien DM (2007). Age-related variation in red blood cell stable isotope ratios (delta13C and delta15N) from two Yupik villages in southwest Alaska: a pilot study. Int J Circumpolar Health.

